# READY, SET, VR!: VOLUNTEERS’ PERCEPTIONS USING VIRTUAL REALITY WITH PLWD IN COMMUNITY-BASED RESPITE

**DOI:** 10.1093/geroni/igae098.0830

**Published:** 2024-12-31

**Authors:** Lillian Agyemang, Mary Milnamow, Jason Dauenhauer, Louanne Bakk

**Affiliations:** University at Buffalo, SUNY, Buffalo, New York, United States; University at Buffalo, Buffalo, New York, United States; State University of New York at Brockport, Brockport, New York, United States; University at Buffalo, SUNY, Buffalo, New York, United States

## Abstract

People living with dementia (PLwD) and their caregivers prefer to “age in place” within the community. Community-based respite centers often have programming that enables caregivers to drop off their care receiver for a few hours while trained volunteers provide engagement. Few, if any, studies have explored Virtual Reality (VR) programming in community-based drop-in respite programs for PLwD. Within this exploratory study, a focus group with 8 volunteers aged 65+ was conducted to understand their experiences delivering VR in a drop-in respite center serving PLwD. Qualitative analysis revealed four primary themes: 1) Motivation, 2) Positive impact of VR, 3) VR Programming, and 4) Enhancing VR delivery. Volunteers shared a strong interest in learning new technology, and their motivation to use VR in respite center programming increased after training. Volunteers observed the positive impact of VR on client engagement and social connections and shared practical tips for improving VR delivery. Results provide evidence that VR can be successfully implemented in a community-based drop-in respite center for PLwD. Structural and individual-level factors may contribute to successful VR implementation, such as programs having technical and financial support from a larger social service agency, and volunteers’ motivation to receiving VR training. The level of enthusiasm demonstrated by volunteers in learning VR contradicts a persistent ageist myth regarding older adults and technology. In conclusion, VR is a novel approach for volunteers and clients to have meaningful social experiences within drop-in respite centers.

